# 1384. Verbal Autopsy as a Tool to Identify Probable Cause of Death in People with Acute Febrile Illness in Zambia — January 2020-December 2022 

**DOI:** 10.1093/ofid/ofad500.1221

**Published:** 2023-11-27

**Authors:** Katie R Hooker, Priscilla Kapombe, Rachel Silver, Chandler Ciuba, Mweene Cheelo, Saviour Moyo, Lloyd Mulenga, Carol Y Rao, Jonas Hines

**Affiliations:** CDC/GHC/DGHP, Tallahassee, Florida; Zambia Ministry of Health, Lusaka, Lusaka, Zambia; CDC/GHC/DGHP, Tallahassee, Florida; Centers for Disease Control and Prevention, Atlanta, Georgia; Zambia Ministry of Health, Lusaka, Lusaka, Zambia; Zambia Ministry of Health, Lusaka, Lusaka, Zambia; CDC/GHC/DGHP, Tallahassee, Florida; CDC/GHC/DGHT, Lusaka, Lusaka, Zambia

## Abstract

**Background:**

Acute febrile illness (AFI) surveillance improves a country’s understanding of what diseases are circulating. However, fever as an entry point for patient identification casts a wide net that will likely capture non-infectious causes of AFI. We used mortality surveillance data to examine probable causes of death (CoD) potentially associated with AFI to expand our understanding of febrile illness in Zambia.

**Methods:**

The Zambian Ministry of Health provided verbal autopsy (VA) data from 27 (of 117) districts representing ∼50% of Zambia’s population. The World Health Organization’s standardized tool for VA was used by hospital staff to interview relatives or close associates of deceased persons of all ages on clinical presentation and circumstances prior to death. The InterVA-5 validated computer algorithm provided a CoD. Using questionnaire data, we applied an AFI case definition based on common global AFI surveillance criteria: a febrile person with a fever duration of < 1-14 days and a mild, moderate, or severe fever classification (in lieu of a temperature cutoff). We analyzed the CoDs and SARS-CoV-2 infections among those that met our AFI case definition in R.

**Results:**

From January 2020 to December 2022, 51,751 VAs were conducted on people over 28 days old–14,503 (28%) reported fever prior to death, of which 12,071 (23%) met our AFI case definition. Broadly, we found that 64% of those that met our AFI case definition had an infectious CoD, 32% had a non-communicable CoD, and 4% had other CoDs. Specifically, the most reported CoDs were acute respiratory infection (14%), diarrheal disease (11%), and HIV/AIDS (10%). A positive SARS-CoV-2 test was reported in 965 (8%) persons with AFI versus 1,876 (5%) without AFI (*p*< 0.001).

**Conclusion:**

AFI-associated deaths were primarily attributable to infectious disease, as identified through VA. AFI is one approach for multi-pathogen surveillance that provides valuable insight into circulating diseases, including SARS-CoV-2, but findings are limited by which pathogens are targeted for testing. Mortality surveillance is a valuable supplementary data source when attempting to understand inconclusive findings in AFI surveillance and aids in a country’s understanding of common causes of febrile illness not captured through AFI surveillance diagnostics.

**Disclosures:**

**All Authors**: No reported disclosures

